# Seasonality of cholera in Kolkata and the influence of climate

**DOI:** 10.1186/s12879-023-08532-1

**Published:** 2023-09-02

**Authors:** Debbie Shackleton, Theo Economou, Fayyaz Ali Memon, Albert Chen, Shanta Dutta, Suman Kanungo, Alok Deb

**Affiliations:** 1 College of Engineering, Mathematics, and Physical Sciences, University of Exeter, Exeter, EX4 4QF UK; 2 Department of Mathematics, University of Exeter, Exeter, UK; 3 Climate and Atmosphere Research Centre, The Cyprus Institute, Nicosia, Cyprus; 4 National Institute of Cholera and Enteric Diseases, Kolkata, India

**Keywords:** Generalized additive modelling, Climate, Cholera, Cross-correlation lag analysis, India

## Abstract

**Background:**

Cholera in Kolkata remains endemic and the Indian city is burdened with a high number of annual cases. Climate change is widely considered to exacerbate cholera, however the precise relationship between climate and cholera is highly heterogeneous in space and considerable variation can be observed even within the Indian subcontinent. To date, relatively few studies have been conducted regarding the influence of climate on cholera in Kolkata.

**Methods:**

We considered 21 years of confirmed cholera cases from the Infectious Disease Hospital in Kolkata during the period of 1999–2019. We used Generalised Additive Modelling (GAM) to extract the non-linear relationship between cholera and different climatic factors; temperature, rainfall and sea surface temperature (SST). Peak associated lag times were identified using cross-correlation lag analysis.

**Results:**

Our findings revealed a bi-annual pattern of cholera cases with two peaks coinciding with the increase in temperature in summer and the onset of monsoon rains. Variables selected as explanatory variables in the GAM model were temperature and rainfall. Temperature was the only significant factor associated with summer cholera (mean temperature of 30.3 °C associated with RR of 3.8) while rainfall was found to be the main driver of monsoon cholera (550 mm total monthly rainfall associated with RR of 3.38). Lag time analysis revealed that the association between temperature and cholera cases in the summer had a longer peak lag time compared to that between rainfall and cholera during the monsoon. We propose several mechanisms by which these relationships are mediated.

**Conclusions:**

Kolkata exhibits a dual-peak phenomenon with independent mediating factors. We suggest that the summer peak is due to increased bacterial concentration in urban water bodies, while the monsoon peak is driven by contaminated flood waters. Our results underscore the potential utility of preventative strategies tailored to these seasonal and climatic patterns, including efforts to reduce direct contact with urban water bodies in summer and to protect residents from flood waters during monsoon.

**Supplementary Information:**

The online version contains supplementary material available at 10.1186/s12879-023-08532-1.

## Background

Cholera is an infectious diarrheal disease caused by contamination with pathogenic strains (O1 or O139) of the bacteria *Vibrio cholerae*. While the disease has been successfully eradicated in many parts of the world thanks to major improvements to drinking water and sanitation infrastructure, the seventh global cholera pandemic persists in many others such as India. Bordering the Bay of Bengal, often considered to be the ‘homeland’ of cholera, the Indian State of West Bengal experiences the highest cholera burden in the country [1], much of which is concentrated in the densely populated state capital Kolkata.

Cholera is highly climate-sensitive and is broadly considered to be exacerbated by the effects of climate change [2–4]. This is particularly true with regards to the endemic cholera which persists across the Bengal Delta, a vast river delta surrounding the Bay of Bengal consisting of Bangladesh in the East, and West Bengal in the West. Transmission in this region occurs predominately due to contact with contaminated water sources in the environment [5]. Within Bangladesh, the relationship between cholera and climate has been studied intensively [6]. An interesting, and potentially unique phenomenon with endemic cholera in this region is the dual-peak seasonality of cases. This is well documented in Bangladesh which experiences peaks both pre- and post-monsoon with a marked abated occurrence of cases during the monsoon itself [7–9]. Within the context of Bangladesh, association between climate and cholera appears to differ between peaks. With regards to rainfall, while the post-monsoon peak has been demonstrated to hold a strong positive association with rainfall [7, 9, 10], during the summer the inverse is true with drier years tending to lead to stronger early peaks [7, 11]. An even more complex relationship appears to exist between cholera and sea surface temperature (SST) in the Bay of Bengal. While evidence gathered from historical cholera mortality records (1891–1940) across the Bengal Delta found coastal SST was positively associated with pre-monsoon cholera, no relationship was found with post-monsoon cholera [8]. In contrast a more recent study in Bangladesh [10] found a weak association coastal SST pre-monsoon, but a much stronger association post-monsoon. The effects of air temperature in this region have been less well considered, but a 2018 study found a significant increase in cholera risk following a heatwave on wet days, but not dry days [12]. Research in other regions has suggested that nonlinear relationships may be present between cholera and climate variables. For example, non-linear relationships have been found between rainfall and cholera in Yemen [13], and between *Vibrio Cholerae* abundance and SST in the North Atlantic [14].

Compared with the rich epidemiological research background of cholera in Bangladesh however, research into the environmental influencers of Kolkata is much more limited despite the presence of endemicity in the region. Geographically, the city of Kolkata is similar to the Bangladesh capital Dhaka where much of the research has been focussed. At a distance of 250 km apart, both are dense urban centres north of the Bay of Bengal with large slum populations who share the same language and many similar cultural practices. However, a study by de Magny et al. [15] has shown that despite their similarities and physical proximity, the influence of climate on cholera in Bangladesh and Kolkata is markedly different.

To date, research conducted into the relationship between cholera and climate in Kolkata has largely been limited to case studies describing particular outbreaks [16], or communities [17]. De Magny et al. [15] used a generalized linear model to assess the effects of rainfall and ocean temperature and chlorophyll levels on cholera in Kolkata. They found a significant influence of rainfall and ocean chlorophyll however only five years of data were used due to limited data available at the time. Significant research gaps remain in clarifying this relationship. Within the Bengal region, the potential presence of nonlinearity in cholera-climate associations has not been ascertained. Within the more specific context of Kolkata, no studies have yet considered the influence of ambient temperature, nor have individual relationships by season have not been considered.

In this study, we aim to fill these gaps by using Generalized Additive Modelling (GAM) to analyse the potentially nonlinear relationships between cholera and three environmental variables: temperature, rainfall, and sea surface temperature (SST) during the period 1999–2019. We also take a closer look at lag times with cross-correlational lag analysis. An additional novel aspect in this study is comparing the use of rainfall runoff with rainfall as a variable. By considering runoff explicitly, we aim to consider the role of contamination of water sources via rainfall runoff in transmission. Finally, we will consider the influence of climate variables on both seasons separately to capture any season-specific relationships.

## Methods

### Study area

**Fig. 1:**
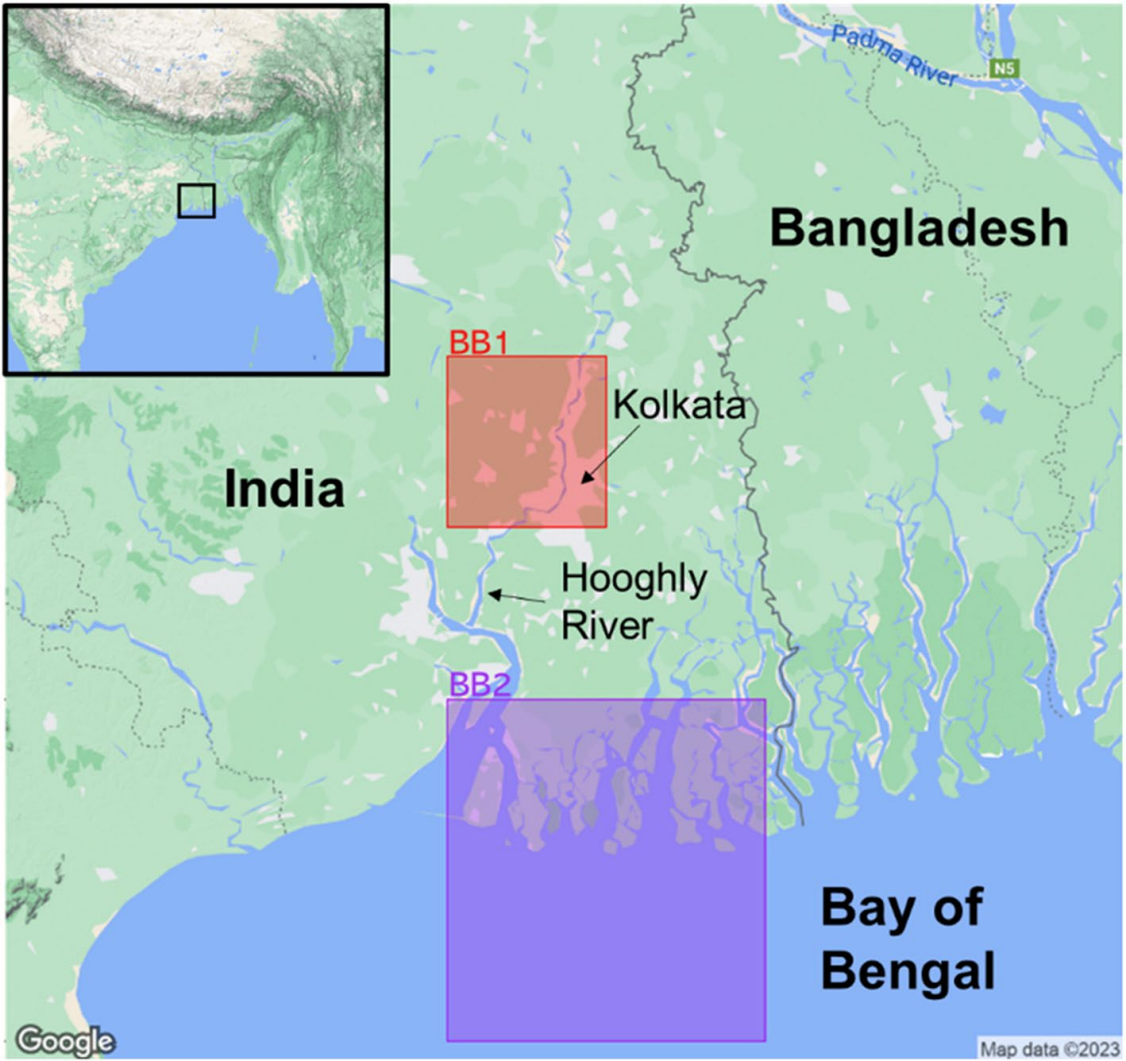
Map of study area showing regions where environmental data were obtained. Bounding box 1 (BB1) shows the 0.5 × 0.5 degree grid cell centred on (88.25E, 22.75 N), bounding box 2 (BB2) denotes the 1 × 1 degree grid cell centred on (88.5E, 21.5 N)

The city of Kolkata lies on the east bank of the Hooghly River, a major Ganges distributary, around 160 km North of its origin into the Bay of Bengal (Fig. 1). It has a total population of around 45 million residents, including many who live in tightly crowded urban slums. The city has an average population density of 24,306 persons per km^2^.

### Epidemiological data

A dataset of stool samples from diarrhoeal patients reporting to the Infectious Disease Hospital (IDH) in Kolkata under their diarrhoeal surveillance system during the 21-year period 1999–2019 was obtained from the Indian Council of Medical Research - National Institute of Cholera and Enteric Diseases (ICMR-NICED). In the surveillance system, every fifth patient on two randomly selected days of the week (representing around 6% of total patients) was tested for several pathogens including O1 and O139 *Vibrio cholerae.* We extracted the number of samples which tested positive for either O1 *or* O139 *Vibrio cholerae* where the patient was registered as residing within the Kolkata Municipal Corporation (KMC) region. The dataset from 1999 to 2007 was un-digitized and pre-aggregated at a monthly resolution. We therefore considered two datasets: a 21-year monthly dataset from 1999 to 2019, and a 12-year dataset (2008–2019) aggregated weekly. The monthly dataset was normalised to a 30-day month to account for differences in month length (Eq. 1).

1 $${case}\,{{s}_{{normalized,month}}} = {case}\,{{s}_{{raw,month}}} \cdot \frac{30}{{days.in.month}}$$

The decadal census populations of Kolkata were recorded as 4,399,819, 4,572,876, and 4,496,694 during the years 1991, 2001, and 2011 respectively (data were unavailable for 2021) [18]. We therefore considered the population of Kolkata to be relatively stable, and therefore ignored any marginal fluctuations in population in our analysis.

### Climate data

Monthly mean daytime temperature (°C) and total rainfall (mm/month) from were obtained from satellite estimations produced by the Climate Research Unit (CRU) at the University of East Anglia from the CRU TS 4.04 dataset [19] (available at http://www.cru.uea.ac.uk/cru/data/hrg/). Rainfall runoff (kg m^−2^ s^−1^) was estimated using the mean monthly output of the following five land-surface models: DLEM [20], ISAM Trendy [21], JULES 1.0 [22], LPX-BERN [23], and ORCHIDEE [24]. We further estimated weekly mean daytime temperature (°C) and total precipitation (mm/day) by extracting daily estimations provided by the CRU Japanese Reanalysis (CRU JRA – available at https://catalogue.ceda.ac.uk/) [25] and aggregating weekly. Each dataset was available at a 0.5 × 0.5 degree spatial resolution and the cell with centroid longitude = 88.25°E, latitude = 22.75°N was chosen since it covered the majority of the KMC area (BB1 in Fig. 1). Monthly SST data (°C) at a 1°x1° resolution were extracted from the Hadley Centre Global Sea Ice and Sea Surface Temperature (HadISST) dataset [26] which combines in-situ and adjusted satellite measurements. We use the grid cell centred at 88.5°E, 21.5°N which refers to the area where the Bay of Bengal meets the mouth of the Hooghly River (BB2 in Fig. 1).

### Statistical analysis

#### Identification of seasonality

A box plot of monthly cholera cases, temperature, and rainfall was produced for visual inspection of seasonality within the monthly dataset. Two distinct ‘cholera seasons’ are demarcated through visual inspection, where the season period is defined according to patterns in cholera cases rather than meteorological patterns.

#### Nonlinear climate relationships

To allow for the potential presence of non-linear associations in the data, we explored the association between cholera cases and environmental variables using Generalized Additive Models (GAMs) [27] applied to the 1999–2019 monthly dataset [27]. The GAM analysis was conducted using the R package *mgcv* [28]. A more in-depth explanation of GAM models is given in the Supplementary materials and is briefly summarized here. GAMs can be considered as an extension to generalized linear models (GLMs) which relaxes the linearity assumption and allows for significantly more flexible model fitting. Here, we model the cholera case data as sampled from a negative binomial distribution (due to the count nature and presence of over-dispersion) where the mean (count) is characterized as the sum of smooth functions of the environmental variables. We considered the variables rainfall, modelled rainfall-runoff, temperature and coastal SST as environmental inputs to the model.

The smooth functions in our model were constructed using penalized cubic regression splines. The flexibility of the smooths can be tuned by increasing the number of knots (k) where a greater number of knots permits a more flexible smooth. Since overfitting is prevented by a smoothing penalization term, we set k to the minimum value above which the influence on the results was negligible to allow sufficient degrees of freedom to describe the true relationship within a manageable computational cost.

In our model, we include the season as a factor within each smooth of an environmental variable which permits the smooths to ‘interact’ with the season factors and produce a particular smooth for each environmental variable in each cholera season. Here, to prevent any potential misinterpretation of environmental correlations as incidental seasonal patterns such as those that may be caused by seasonal migration or major annual festivals, we include a smooth for month using a cyclic penalized cubic regression spline. Similarly, to account for long-term trends not caused by environmental changes (e.g., due to improvements to sanitation or changes in health seeking behaviour) we include a smooth function of date.

To reduce bias linked with assumptions about lags in the connection between environmental variables and cholera, we analysed five different lag configurations for each of the four climate variables, as well as the option of omitting each variable. These configurations were as follows: variable omitted, in sync with the cholera occurrence month, delayed by one month, delayed by two months, mean average of concurrent and one-month lag, mean average of a one-month and a two-month lag. This examination led to a total of $N={6}^{4}-1=1296$ model variations. The general formula for each GAM model is given in Eq. 2.

2 $${log}\left[ E(Y_{m}) \right] = \beta_{0} + s_{season}(temperature_{m,lag}) + s_{season}(rainfall_{m,lag}) + s_{season}(runoff_{m,lag}) + s_{season}(SST_{m,lag}) + s(date) + s(month) + season$$

Where $E\left({Y}_{m}\right)$ represents the expected monthly confirmed cholera cases in month $m$, ${\beta }_{0}$ represents the intercept, and ${s}_{season}\left(\right)$ represents the smooth functions within a particular cholera season.

The strength of each model was assessed by calculating the Akaike Information Criterion (AIC) [29] which measures goodness-of-fit while penalizing additional model complexity. The model with the lowest AIC score was selected as the final model.

We anticipated temporal autocorrelation in the counts, and after exploratory analysis of the autocorrelation function (ACF) and the partial ACF (PACF) of the residuals of the chosen model (Figure S1), an AR(1) process was indicated. We therefore included an additional one-month lagged term of the response variable (as a linear covariate with its own associated Beta coefficient) in the final model (Eq. 3). This inclusion of an autoregressive term to deal with autocorrelation in the data is a well-established approach [30]. An ACF plot of the final model (Figure S2) confirmed this to be a reasonable assumption.

3 $$log\left[E\left(Y_m\right)\right]=\beta_0+s_{season}\left(temperature_{m,lag}\right)+s_{season}\left(rainfall_{m,lag}\right)+s_{season}\left(runoff_{m,lag}\right)+s_{season}\left(SST_{m,lag}\right)+s\left(month\right)\;+\;s\left(month-of-year\right)+season+log\left[y_{m-1}\right]$$

#### Lag analysis

We next used cross-correlation analysis to identify the lags associated with the strongest relationship between climate variables and cholera cases. Specifically, we measured the Pearson’s correlation between climate and cholera time series at lag periods from 0 to 25 weeks stratified by season. We utilised the 12-year weekly aggregate datasets from 2008 to 2019 to assess the effect of lag times at a finer temporal resolution.

### Limitations

In our study we conclude that the relationship observed between rainfall and cholera during the monsoon season is due to urban flooding. However, measuring rainfall-induced flooding using monthly averages does not fully capture tropical cyclones and short periods of highly intense rainfalls responsible for the flash flooding which frequently occurs in Kolkata [31]. It is therefore possible that the associations with rainfall found in this study are underestimated. Another issue in this study is the uncertainty in the cholera case data. Only cholera patients who report to the ID hospital in Eastern Kolkata may be included in the confirmed cases. Therefore, reported case numbers are highly sensitive to changes in health-seeking behaviour. It is possible, for example, that during periods of heavy rains typically associated with high cholera risk, residents may be more encouraged to seek hospital treatment. Further, as only around 6% of ID hospital patients are selected for testing, the total number of confirmed cases is low, with an average of only around 10 per month. This makes the data highly susceptible to random noise which could potentially mask statistically significant relationships.

## Results

### Descriptive statistics

**Fig. 2:**
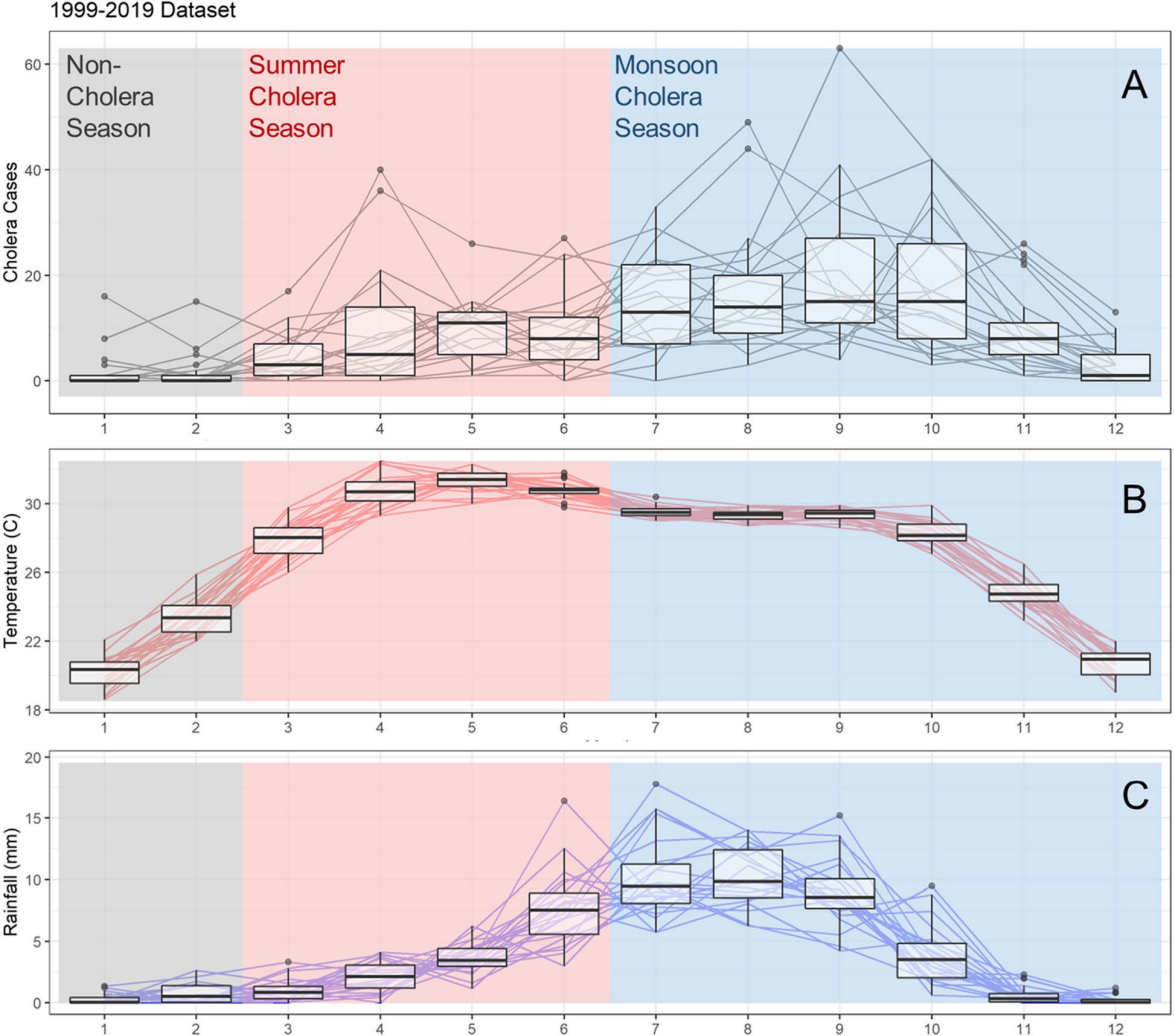
Variation in monthly values between 1999–2019 for **A** confirmed cholera cases in Kolkata, **B** mean daytime temperature in the Kolkata area, **C** total precipitation in the Kolkata area. Each line represents values for one year. Boxplots are overlaid to display mean and interquartile ranges for each month. Background colour represents cholera season demarcation, non-cholera season is shown as grey, red as summer, and blue as monsoon

A total of 2479 confirmed cases of cholera were recorded between 1999 and 2019, the time series is given in Figure S4. To detect the presence of seasonality within cholera cases, we plotted a boxplot of total cholera cases per month from 1999 to 2019 (Fig. 2A). A subtle but distinct bi-annual pattern can be witnessed in the monthly data, the first peaking in April/May with a slight lull in June, followed by a larger peak around September. It can further be seen from that the first peak coincides with an increase in temperature as summer approaches (Fig. 2B), and the second after the onset of Monsoon rains (Fig. 2C). We therefore demarcated summer cholera as cases reported during the four-month period March-June, and monsoon cholera as cases reported during the six-month period July-December. January and February are considered as ‘non-cholera’ seasons. Summary statistics for cholera cases, temperature and rainfall are displayed in Table 1.

**Table 1:** Descriptive summary of seasonal cholera cases, temperature, rainfall and runoff during the years 1999–2019 using monthly dataset

	Season	Mean	Min	Max	Standard Deviation
Monthly Cases	total	9.84	0	63	10.54
	monsoon	13.71	0	63	11.68
	summer	8.11	0	40	8.04
	non-cholera	1.69	0	16	3.61
Temperature (°C)	total	27.20	18.60	32.50	3.83
	monsoon	27.00	19.00	30.40	3.31
	summer	30.20	26.00	32.50	1.48
	non-cholera	21.80	18.60	25.90	1.89
SST (°C)	total	27.64	22.02	30.87	2.29
	monsoon	28.20	23.92	30.11	1.76
	summer	28.73	25.82	30.87	1.27
	non-cholera	23.76	22.02	25.63	0.81
Rainfall (mm/month)	total	126.8	0.00	551.6	132.6
	monsoon	175.3	0.00	551.6	150.2
	summer	110.2	0.00	491.8	93.7
	non-cholera	14.4	0.00	74.1	18.9
Runoff (kg m^−2^ s^−1^)	total	16.92	0.66	119.04	23.66
	monsoon	29.11	1.81	119.04	27.86
	summer	6.19	0.66	59.09	8.12
	non-cholera	1.83	1.02	4.67	0.80

### GAM analysis

The model variation with the lowest AIC score (AIC = 1312.3) and selected for analysis retained temperature and rainfall as explanatory variables but omitted SST and rainfall runoff (Eq. 4). The selected lag for temperature was the mean average of the concurrent and previous month, no lag was chosen for rainfall and its concurrent value was selected. The model was able to explain 55.5% of the deviance in the data and residual analysis (Figure S3) suggests model assumptions are reasonable.

4 $$log\left[E\left(t\right)\right]=\beta_0+s_{season}\left(temperature_{m,lag=mean\left(0,1\right)}\right)+s_{season}\left(rainfall_{m,lag=0}\right)+s\left(month\right)+s\left(month-of-year\right)+season+log\left[y_{t-1}\right]$$

**Fig. 3:**
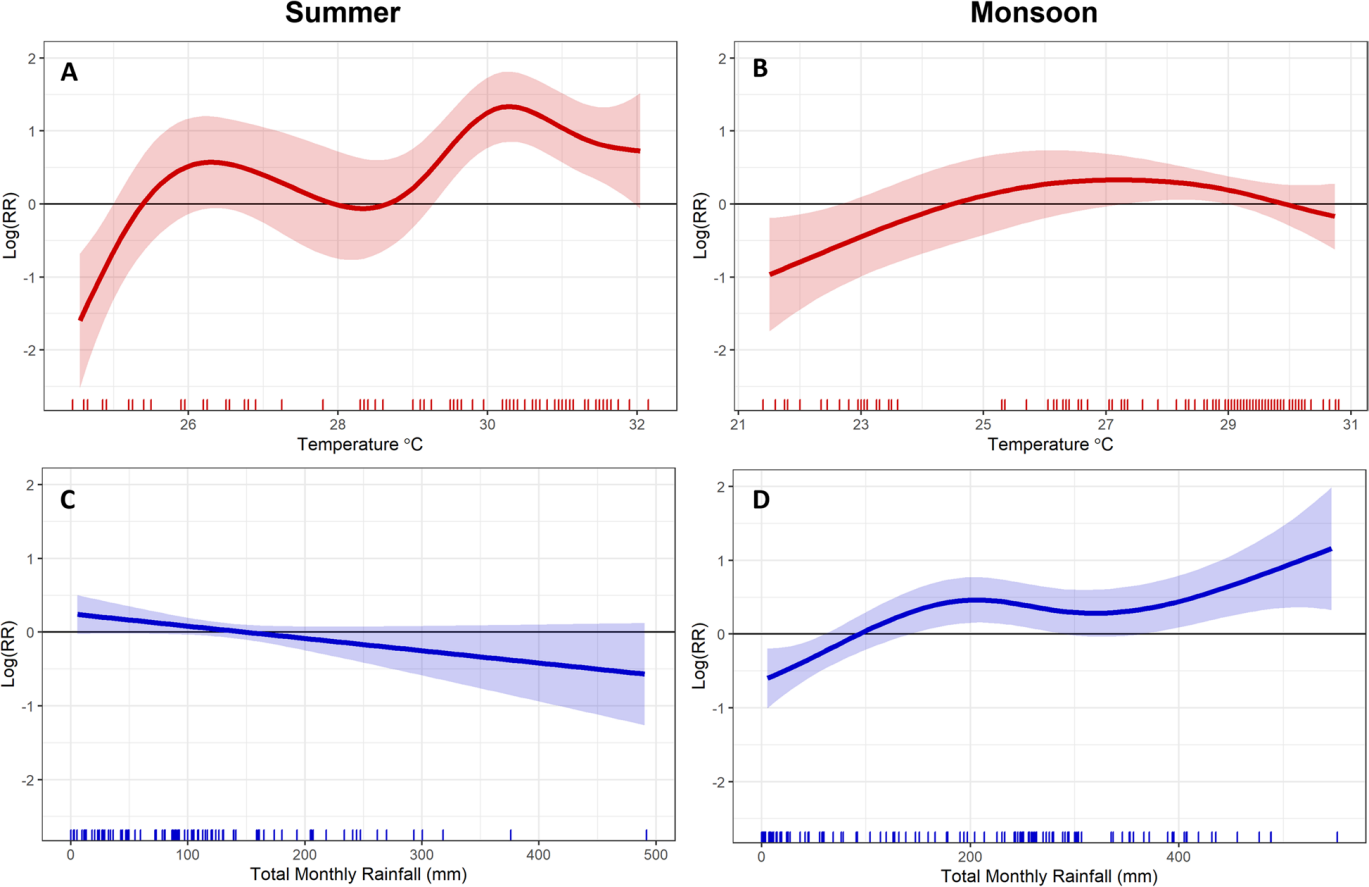
Partial effect curves for relationship between reported cholera cases with average temperature across 0- and 1-month lag (**A**, **B**) and rainfall in concurrent month (**C**, **D**) in summer(left) and monsoon (right) seasons after controlling for seasonal and long-term trends and the inclusion of a linear autoregressive covariate. The 95% confidence interval is shown with the lighter band. The null hypothesis of no effect is shown by the horizontal black line; significant relationships can be inferred in regions where the confidence interval does intersect the black line. The rug plot at the bottom of each plot displays the observed values for each covariate within each season. The y-axis represents the (logged) relative risk of cholera incidence with respect to the (logged) mean of the response variable

The partial effect curves for each variable-season combination are shown in Fig. 3. These represent the component effect of each environmental term in the model which, when combined with the long-term and seasonal partial effects (Figure S5), autoregressive terms and intercept ${\beta }_{0}$, sum to the overall prediction of the model. The y-axis denotes the logged relative risk and can be interpreted as the logged relative expected cholera case count with respect to the seasonal mean.

During the summer season the influence of increased temperature appears greatest at mean temperatures above 29 °C, with a maximum relative risk (RR) of 3.8 (95%CI 2.35–6.13) occurring at 30.3 °C. Temperatures below 25 °C were also associated with reduced risk where a mean temperature of 24.4 °C has an estimated RR of 0.16 (95%CI 0.06–0.45). From 3 C it appears there is a slightly negative association between monthly rainfall and cholera cases, however this relationship is not statistically significant.

During monsoon season, the influence of temperature was considerably less pronounced with only temperatures below 22.7 °C being significantly associated with reduced RR, where RR is estimated at 0.37 (95%CI 0.16–0.82) at 21.4 °C. However, the effect of rainfall was much more influential. A total monthly rainfall of 550 mm was associated with an RR of 3.38 (95%CI 1.37–7.86). It can be seen from Fig. 3 that all three signficant relationships are considerably non-linear.

An overall linearly decreasing trend beginning from 2010 can be witnessed in the smooth for long-term trends (Figure S5A). Interestingly the model has estimated a null seasonal trend (Figure S5B) which suggests that the seasonal patterns observed in the data were fully explained by the environmental smooths.

### Lag times

**Fig. 4:**
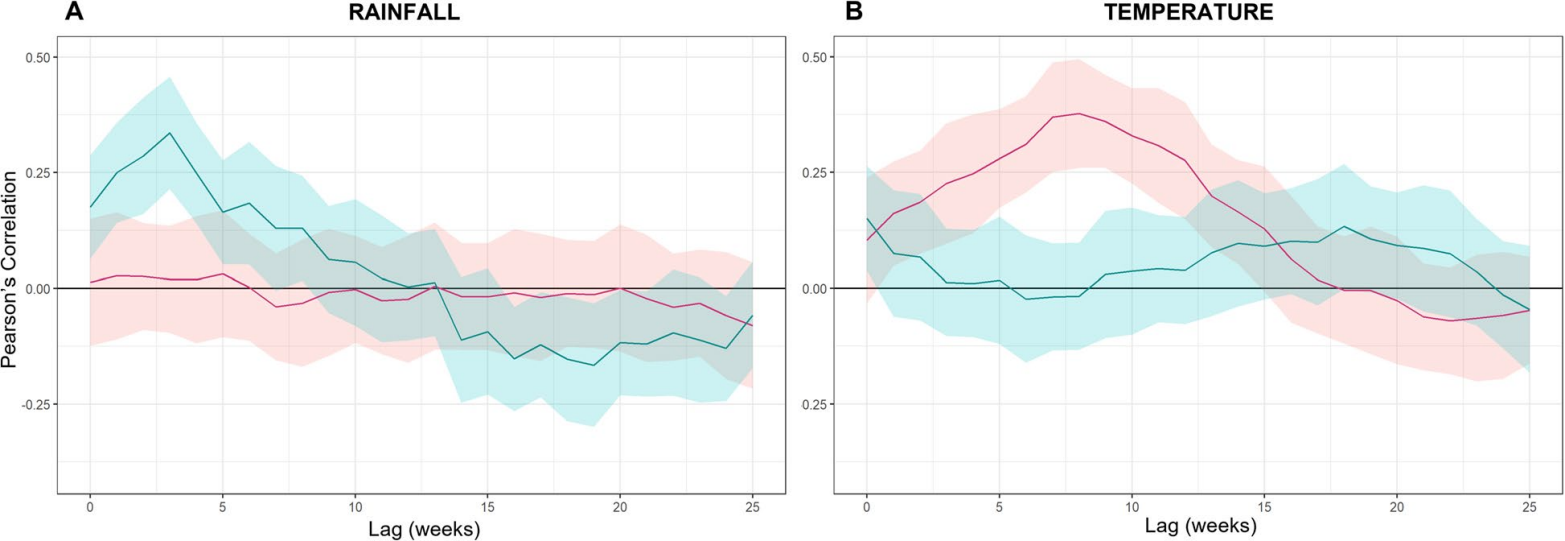
Correlation between aggregated seasonal cholera and concurrent/lagged seasonal rainfall (**A**) and temperature (**B**) during summer (red) and monsoon (blue) for lags 0–25 weeks. Lighter band represents the 95% confidence interval

We computed the strength of the correlation between rainfall and temperature time series with the weekly cholera case time series at lags from 0 to 25 weeks (Fig. 4 )*.* We found that the rainfall was significantly and positively correlated with cholera cases during the monsoon season at lags from 0 to 8 weeks with the strongest association occurring at 3 weeks lag. Interestingly a smaller but still significant negative correlation is witnessed from lags 16–21 weeks. Rainfall was not correlated with cholera cases during summer at any of the lags considered. Conversely, temperature was positively associated with cholera cases during the summer season from lags 1–13 weeks, peaking at 7 weeks.

## Discussion

In this study, we explored the relationship between climate and cholera in Kolkata. We determined that cholera in Kolkata demonstrates bi-annual seasonality with distinct summer and monsoon peaks. Further, we found that there is a difference in the factors which influence the magnitude of summer and monsoon cholera peaks; while temperature is the main driver of summer cholera, rainfall dominates the relationship with monsoon cholera. The model which best fit the data did not include SST or rainfall runoff, suggesting that neither variable is helpful in explaining the relationship between climate and cholera. We also found the peak associated lag time between temperature and cholera cases in the summer was more than double that of peak lag time between rainfall and cholera during monsoon.

The seasonal cholera pattern we found in Kolkata is similar to that found in Bangladesh, though with a less pronounced inter-peak period with the secondary peak beginning during the monsoon rather than following it [7]. However, the pattern is markedly different to that recorded in a historical dataset of the region (then known as Calcutta) during 1891–1940. During that period, the city experienced a single large peak occurring during the summer [8]. We suggest the following explanation for the change in single summer peak cholera in the early 1900s to the dual-peak pattern with maximum during the monsoon observed in our study. A reported 100% of residents of modern (2020) Kolkata have some form of access to a treated municipal water supply [32] meaning that their exposure to the multiplying *Vibrio cholerae* in urban water bodies is likely to be considerably reduced compared with the period 1890–1941, and thereby lessening the potency of this mechanism and reducing the magnitude of the summer peak. While access to sanitation in the region is likely to have increased significantly over the past 100 years, the proportion of households with an improved sanitation facility was only 60.9% and 48.4% in 2019 and 2015 respectively [32] indicating that residents remain vulnerable to the effects of flood water mixing with raw sewage during the monsoon. We propose that a combination of lower immunity levels (due to the lessened summer peak) and greater vulnerability to flood waters due to higher population density (around 4x greater in 1991 compared with 1911 [33]) could have introduced the presence of a monsoon peak.

The difference in associated climate factors between summer and monsoon cholera suggests distinct mechanisms mediating cholera transmission in each season. A potential explanation for the strong association between temperature and summer cholera witnessed in our study is that urban surface water is an important transmission route early in the year. Kolkata residents often come into contact with urban water sources such as ponds, rivers and lakes for the purposes of washing, bathing and swimming [34–36].

We consider that there are two potential explanations for the association with temperature. The first, as posited by Akanda et al. [7] among others, is that the preference of *V.cholerae* for warmer waters leads to proliferation of the bacteria during hot periods. This is in line with laboratory studies which found that *V.cholerae O1* cells multiply most effectively when incubated at warmer temperatures up to 30 °C [37, 38]. Thus, increased temperatures could lead to an increase in concentration of the pathogenic bacteria and thereby, due to the dose dependent nature of cholera infections [39], increase the probability of cholera infection at a given interaction with urban surface water bodies. A second, alternative explanation for the association between temperature and summer cholera cases is increased contact with water bodies such as ponds, canals and the Hooghly River as one of the few available methods of ‘cooling off’ during heatwaves available to the urban poor. The results of our lag analysis support the former hypothesis as a longer lag time would be expected under such as scenario to allow for a build-up of bacteria in urban water bodies before reaching a critical threshold capable of causing cholera infection. The latter hypothesis would predict a much shorter associated lag time, namely the length the time between a given hot day, and the time taken for cholera to take hold enough for the patient to be admitted to hospital and the stool sample to be taken – a time likely to be no longer than 2–3 weeks.

A further interesting finding with regards to the relationship between cholera and temperature is its marked non-linearity, echoing findings previously reported in Azerbaijan where cholera cases peaked at an air temperature of 25 °C [40]. Similarly, our results show a linear correlation up until approximately 26 °C, after which the trend weakens.

The positive association with rainfall only witnessed in the monsoon season in our study is highly consistent with results from Bangladesh [12, 14]. One potential explanation for the positive association with rainfall, also suggested by Akanda et al. [10] among others, is that rainfall induced floods lead to mixing between flood and sewage waters, as well as an increased contact between residents and contaminated flood waters. An alternative explanation which has been suggested to explain cholera outbreaks in Central India [41] involves the role of rainfall runoff. In areas where open defecation remains an issue, including Kolkata [42], rainfall can wash human faecal matter from these open defecation sites into accessible water sources, leading to contamination and potential cholera outbreaks. However, this second explanation would suggest that modelled rainfall runoff would be a better predictor variable for cholera than rainfall, which was not supported in our study. Further a significant role of contaminated runoff would imply a positive association with rainfall during both dry and wet seasons, which is also inconsistent with our findings. A lack of positive association between cholera and rainfall in the summer remains consistent with the flooding hypothesis, as the rainfall which occurs during the summer generally does not lead to flood events [43]. We therefore suggest that the flooding hypothesis is likely the primary mechanism mediating the monsoon rainfall-cholera relationship.

Increases in SST in the Bay of Bengal have been suggested to be linked to cholera cases in the Bengal Delta by an associated increase in phytoplankton. This is suggested to increase both zooplankton populations and pH levels, both of which are considered to promote *Vibrio Cholerae* populations in local estuaries [6]. Coastal intrusion, especially during the summer, then leads to greater *Vibrio Cholerae* concentrations in the rivers upon which many urban areas are built upon. That our study found SST was not a useful indicator of cholera, highlights the spatial heterogeneity in SST-cholera relationships across the Bengal Delta. The reasons for these discrepancies remain unclear, especially given Kolkata’s proximity to the Hooghly River, which is highly estuarine in Kolkata with a tidal oscillation of ~ 3 m [44], however this could indicate a decreased role of the river in cholera transmission in place of ponds and canals compared with Dhaka.

Our results suggest a mediating role of increased pathogenic *V.cholerae* in urban water bodies and as such efforts to reduce direct contact with urban water bodies, such as through public health campaigns or improved washing facilities preceding and during the summer season could be effective at reducing cholera cases. Conversely, our results suggest a mediating role of contact with contaminated flood waters during the monsoon cholera season, and therefore interventions focussing on protecting residents from flood waters could be most effective in the short-term – and in the long-term, universal safe sanitation to prevent initial contamination of flood waters.

Our findings suggest that hotter summers and wetter monsoons are conducive to high numbers of cholera cases in Kolkata. This is concerning due to a widely projected increase in the volume of rain falling during the monsoon season in the South Asian region a result of greenhouse-gas forcing [45]. In addition summer heatwaves are predicted to become frequent, intense, and prolonged across South Asia with maximum exposure occurring in the Indo-Gangetic Plain [46]. This suggests that, from a climate standpoint, the vulnerability of Kolkata to cholera is likely to increase over time. It is therefore vital to remain vigilant in efforts to improve sanitation in the region.

## Conclusions

Our results find significant non-linear relationships between climate factors and cholera in Kolkata, with temperature driving summer cholera and rainfall driving monsoon cholera. We suggest the summer outbreak is mediated by increased pathogenic *Vibrio* concentration in urban water bodies, and the monsoon outbreak by contaminated rainfall-runoff. In this sense, we find that the relationship with climate in Kolkata is similar to that in Bangladesh. However, important differences were found including a lack of association with SST in Kolkata and a diminished early-monsoon lull. With regards to intervention strategies, we suggest that summer and monsoon peaks are considered separately with an increased emphasis on separation from urban water bodies during the summer season, and protection from flood waters in the monsoon.

### Supplementary Information

**Additional file 1.**

## Data Availability

Rainfall, temperature, and SST data are all publicly available, with details given in the methodology section. Monthly rainfall and temperature data can be accessed at https://catalogue.ceda.ac.uk/ and weekly data at https://crudata.uea.ac.uk/cru/data//hrg/. Monthly SST data can be accessed at https://catalogue.ceda.ac.uk/uuid/facafa2ae494597166217a9121a62d3c. Epidemiological data is the property of the Indian Council of Medical Research (ICMR) and is not publicly available due to political, privacy and ethical concerns. Please contact the corresponding author to request the R code files.
