# Supplementary material for: Seasonality of cholera in Kolkata and the influence of climate
Source: BMC Infect Dis. 2023 Sep 2;23:572. doi: 10.1186/s12879-023-08532-1 (PMC10474634; doi:10.1186/s12879-023-08532-1)
Supplement: Supplementary file 1 — Additional file 1. [file 12879_2023_8532_MOESM1_ESM.docx]

**Supplementary Materials**

1. **GAM Methodology**

**GAMs**

Generalized additive models (GAMs) were first developed by Hastie and Tibshirani 1986 as an effective way of modelling non-linear relationships [24]. They have the benefits of being much more flexible than a linear regression model, but retain much more interpretability than ‘black box’ learning algorithms. The general concept behind GAMs is that the dependant variable $y$, can be estimated by the sum of smooth patterns from individual predictor variables and take the general structure as in Equation S1:

Equation S1

$$g\left( E\left( Y \right) \right)=\alpha+s_{1}\left( x_{1} \right)+s_{2}\left( x_{2} \right)+\ldots+s_{n}\left( x_{n} \right)$$

Where $g()$is some function from the exponential family, $\alpha$ is the intercept, and $s_{1}(),\ldots,s_{n}()$ are smooth nonparametric functions. A variety of smooth functions may be permitted in GAM modelling, however in our analysis we use penalized cubic regression splines due to their relative simplicity and versatility within unidimensional smooths.

**Penalized cubic regression splines**

To describe a penalized cubic regression spline we first begin with the concept of a cubic spline. These are a construction of sections of cubic polynomials and are joined at specified locations known as ‘knots’ such that the curve is continuous up to the second derivative. It follows that the greater the number of knots, the greater the flexibility of the spline. In our analysis we also use a related spline called a cyclic penalized cubic regression spline which extends the concept of a penalized cubic regression spline such that two ends also are continuous up to the second derivative. Cubic spline regression attempts to fit the spline to data by minimizing the residual sum of squares. However, this approach can lead to overfitting and excessive ‘wiggliness’. The solution to this is to reward smoothness by adding a penalization term to the fitting function which calculates the integrated square of the second derivatives (i.e. the slope of the slopes). The fitting function is given in Equation S2

Equation S2

$$\sum_{i=1}^{n} \left( y_{i}-f\left( x_{i} \right) \right)^{2}+\lambda\int\left( f''\left( x_{i} \right) \right)^{2}dx$$

Where the first term in Equation S2 is the standard residual sum of squares, and the second is the penalization term. $\lambda$ is a tuneable smoothing parameter such that as $\lambda\to\infty$ the smooth becomes linear. In the R package *mgcv* [10]optimizes the smoothing parameter by Restricted Maximum Likelihood (REML).

1. **Model Checking**

**
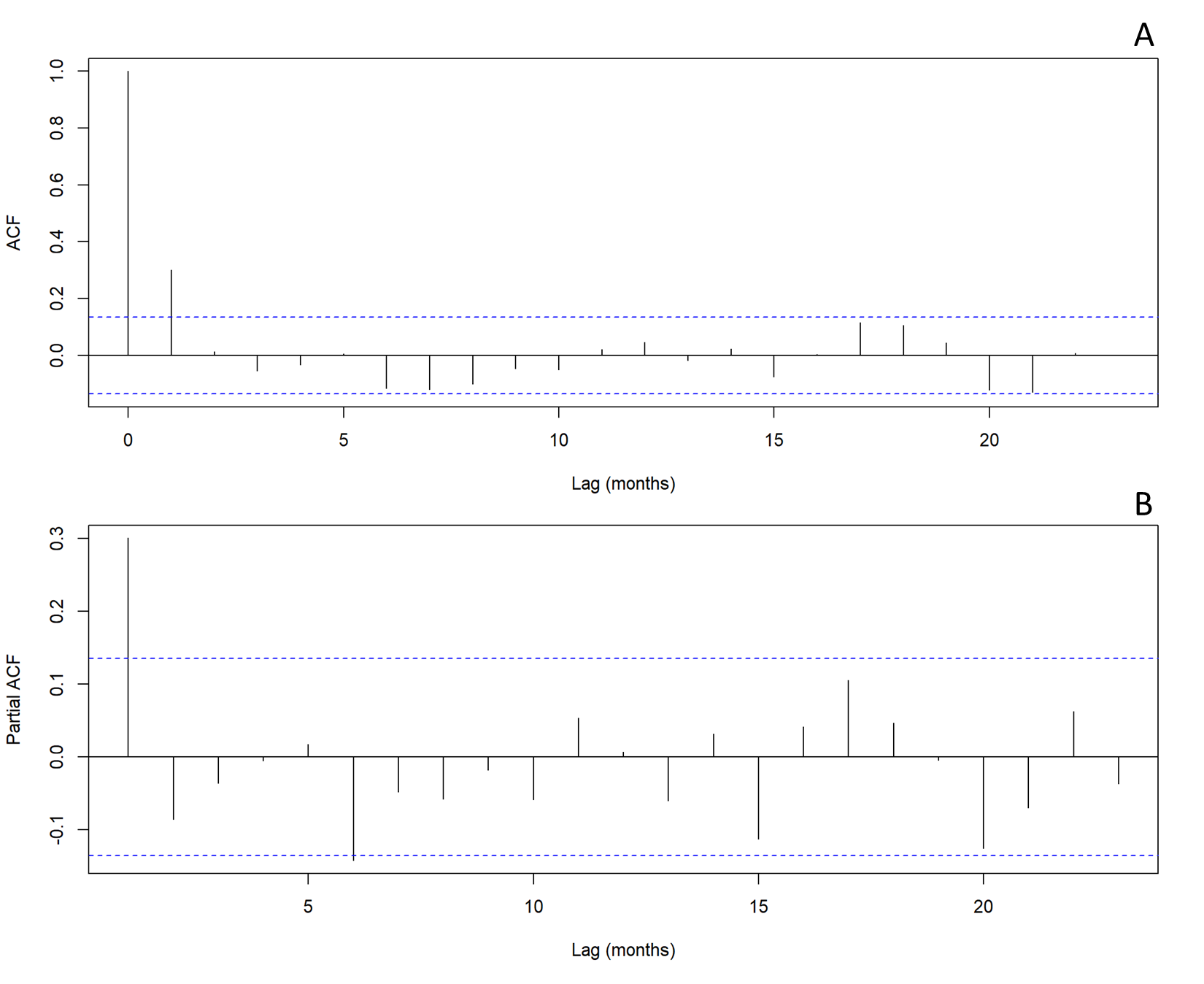
**

Figure S1 – Autocorrelation (A) and Partial Autocorrelation (B) plots of gam model prior to addition of autoregressive terms. Blue dashed line shows level of 95% statistical significance. (A) Demonstrates significant levels of autocorrelation in the model residuals at non-zero lags. From (B), the significant spike a 1 month lag indicates an AR(1) process.


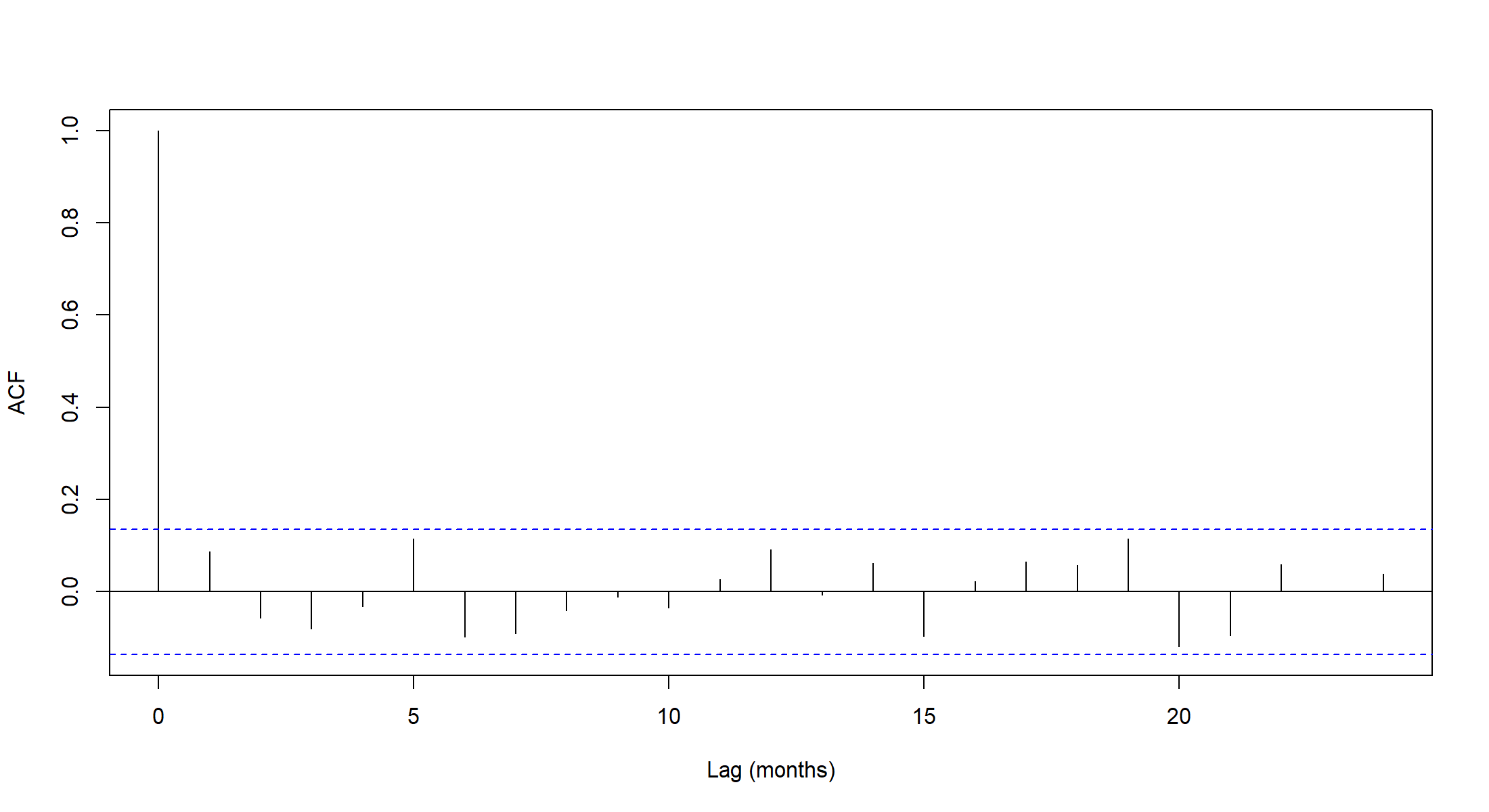


Figure S2 - Autocorrelation of residuals of final GAM model with additional y_t-1­_ term. Blue dashed line shows level of 95% statistical significance. The lack of statistical significance at any non-zero lag period indicates that remaining autocorrelation in the model is of an acceptable level.


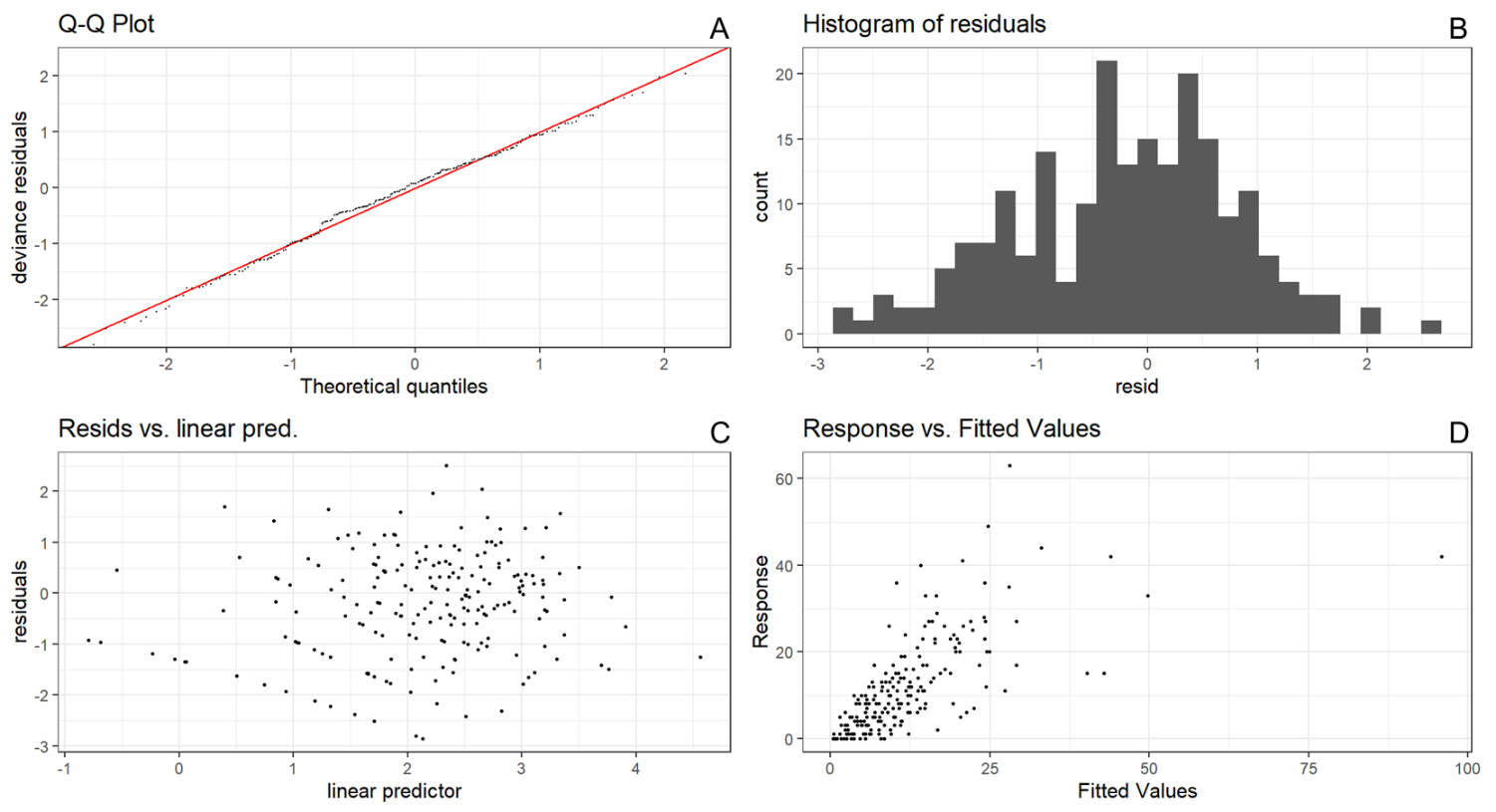


Figure S3. Model checking residual analysis plot of GAM. (A) Shows a Quantile-quantile (QQ) of model residuals against quantiles derived from simulating data from the fitted model. The position of points along the x=y line suggests the modelling distribution assumption (here negative binomial) is reasonable. The histogram of model residuals in (B) roughly follows the normal distribution which does not suggest any underlying assumptions in the model have been violated. (C) Shows a plot of model residuals on the y-axis and predictor values on the x-axis. That the points appear to be symmetrically distributed in both x and y axes supports the validity of the model. (D) Shows the response variable vs. the fitted value. The points mostly follow a diagonal band around the (0,1) line indicating the model fits the data well with one major outlier.

1. **Additional Plots**


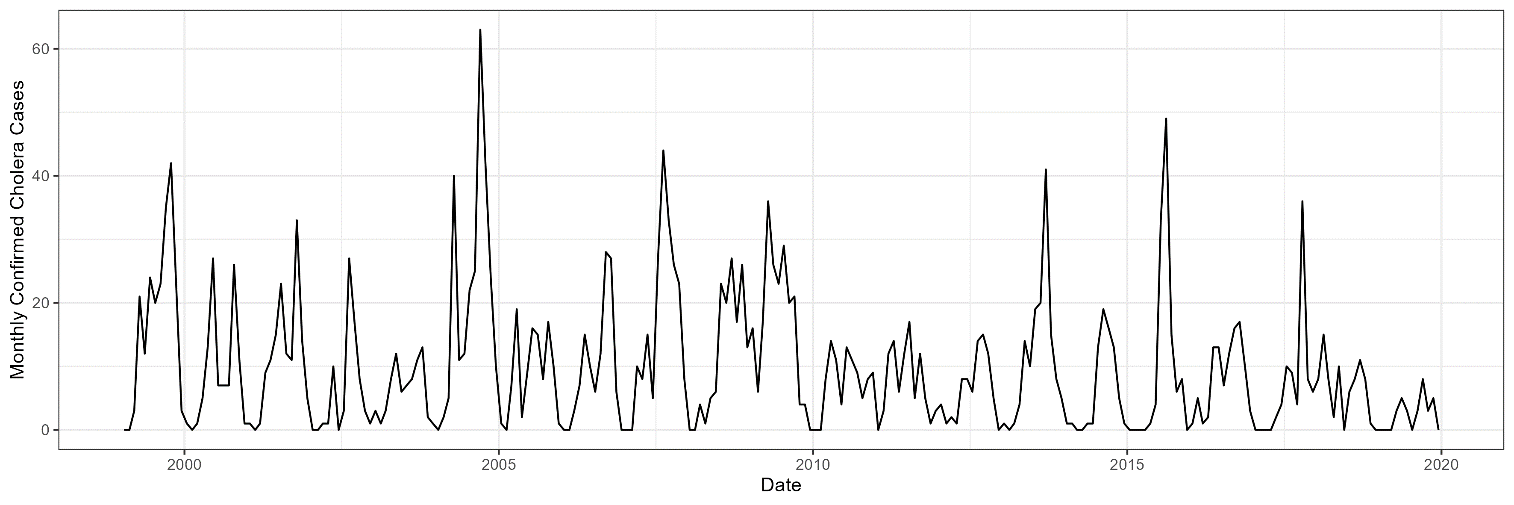


Figure S4 – Time series of confirmed cholera cases recorded in ID Hospital, Kolkata from 1999-2019.


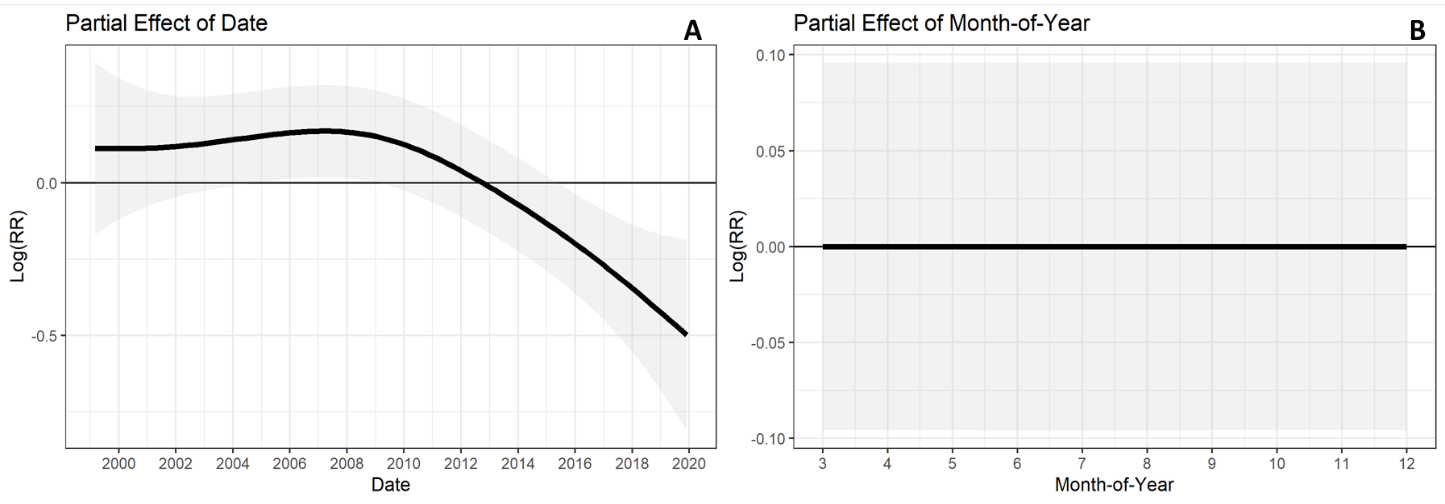

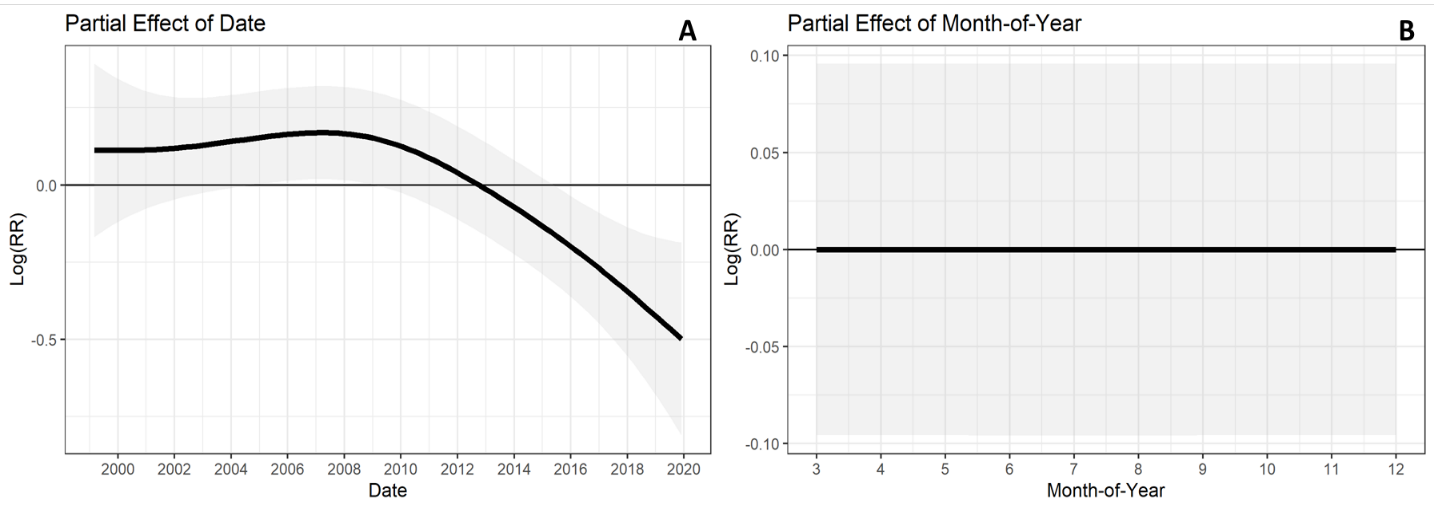


Figure S5 – Partial effect curves for non-environmental smooths in GAM model. The long term trend estimated by the model is shown by the partial effect of date in (A) where a decreasing trend is evident. The seasonal trend is estimated by the model given by the partial effect of month-of-year (B).
